# Nanovibrational Stimulation of *Escherichia coli* Mitigates Surface Adhesion by Altering Cell Membrane Potential

**DOI:** 10.1021/acsnano.4c11000

**Published:** 2024-10-22

**Authors:** Dario G. Bazzoli, Nasim Mahmoodi, Terri-Anne Verrill, Tim W. Overton, Paula M. Mendes

**Affiliations:** School of Chemical Engineering, University of Birmingham, Birmingham B15 2TT, U.K.

**Keywords:** surface adhesion, membrane potential, mechanobiology, bacteria, vibrations

## Abstract

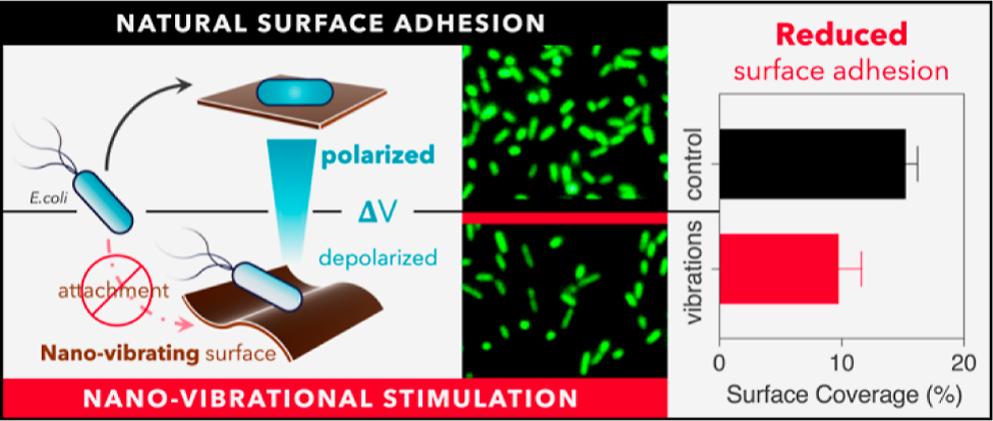

Mechanical forces shape living matter from the macro- to the microscale as both eukaryotic and prokaryotic cells are force wielders and sensors. However, whereas such forces have been used to control mechanically dependent behaviors in mammalian cells, we lack the same level of understanding in bacteria. Surface adhesion, the initial stages of biofilm formation and surface biofouling, is a mechanically dependent process, which makes it an ideal target for mechano-control. In this study, we employed nanometer surface vibrations to mechanically stimulate bacteria and investigate their effect on adhesion. We discovered that vibrational stimulation at the nanoscale consistently reduces surface adhesion by altering cell membrane potential. Our findings identify a link between bacteria electrophysiology and surface adhesion and provide evidence that the nanometric mechanical “tickling” of bacteria can inhibit surface adhesion.

Biofilms are currently a major economical and medical threat to our society because of surface biofouling which could lead to material degradation^1,2^ and chronic infections.^3,4^ Due to the high antibiotic resistance of mature biofilms,^5,6^ recent strategies aim at preventing the seminal step to their formation: surface adhesion. Physical modification of surface’s topology and morphology or its chemical functionalization have been used in the past to mitigate adhesion by either killing or repelling approaching bacteria.^7^ While these physicochemical strategies prove successful to some extent, current draw backs are that they are costly to implement, not all hard surfaces can be functionalized, and their long-term efficacy is undermined by the passive deposition of dead cells or debris and by the depletion of active components. Complementary to these “surface centric” approaches, mechanically targeting cell sensing and physiology could prove another means to suppress surface adhesion.

The ability to sense and respond to mechanical stimuli is ubiquitous across all domains of life from eukarya to bacteria. While our knowledge of mammalian mechanobiology is broader, older, and deeper, the past decade has greatly compensated for our late occurring appreciation of this phenomenon in bacteria. Similar to mammalian cells, mechanosensitive elements and mechanotransduction pathways allow bacteria to detect mechanical cues and use them to regulate surface adhesion.^8^ Multiple mechanosensors and pathways mediate the process in different species, but many of these are unified by the resulting higher levels of the intracellular second messenger cyclic diguanylate monophosphate (c-di-GMP) following surface contact.^9^ Flagella and type IV pili have been reported to act as mechanosensory elements, signaling for adhesion in both *Caulobacter crescentus* and *Pseudomonas aeruginosa*.^10−14^ In *Escherichia coli*, although no clear mechanotransduction pathway has yet been identified, increases in c-di-GMP concentration dependent upon a functional flagellar motor have also been shown to occur soon after surface contact.^15^

Because of this reliance on mechanical sensing, exogenous mechanical cues could control surface adhesion by interfering with cell mechanotransduction, providing the prime technological opportunity to regulate bacterial adhesion through mechanical stimulation.

However, despite our advancements in physically controlling bacterial behavior^16,17^ and the prevalence of mechanical sensing in the microcosm,^18^ our control over it is currently limited to mammalian cells.

Nanovibrating surfaces are effective at controlling mechanically dependent behaviors, such as osteogenesis in mesenchymal stem cells,^19,20^ and represent a promising tool for mechanically stimulating bacteria. Relative to existing strategies such as atomic force microscopy, gel encapsulation, extrusion loading, or cell bending,^21^ nanovibrational stimulation offer the advantage of being applicable to whole surface populations without embedding cells in matrixes or microfluidic devices. Although few attempts have been made at applying vibrational and nanovibrational strategies to bacteria,^22−24^ their impact on single cells’ physiology is unknown. In this work, we used nanometric surface vibrations to “tickle” surface-approaching *E. coli* cells with mechanical cues and investigated their influence on surface adhesion and physiology.

## Results and Discussion

### Vibrational Device Characterization and Estimation of Stimulating Forces

**Figure 1:**
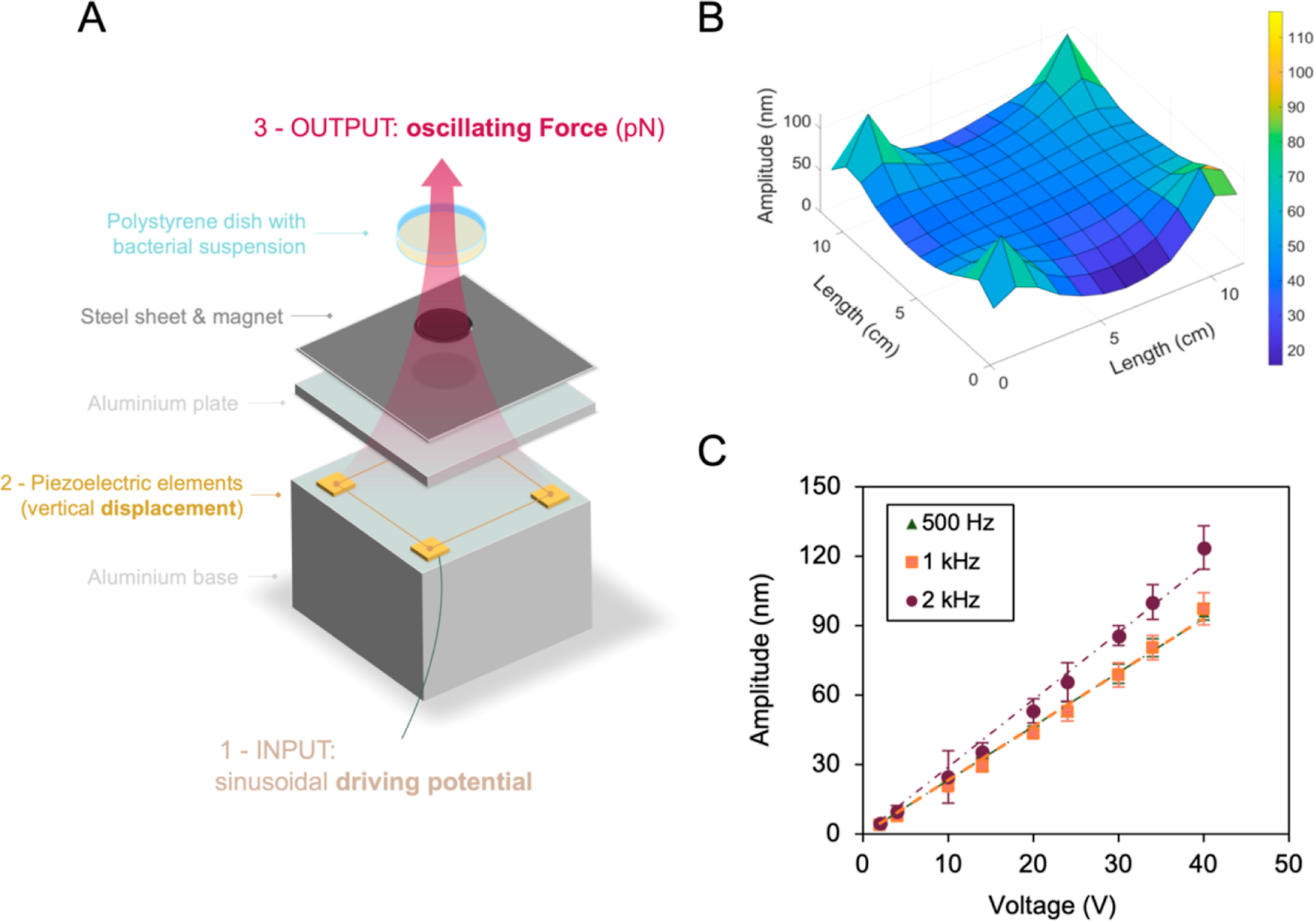
(A) Schematic representation of the vibrational apparatus. From bottom to top, a signal generator and amplifier send an electric input to four piezoelectric actuators whose expansion vibrates a steel covered aluminum plate and a magnetically bound polystyrene Petri dish containing *E. coli* suspensions. (B) Laser vibrometry data of vibrational amplitude from the center of 144 squared tiles (1 × 1 cm^2^) on the steel sheet covering the aluminum plate in A. This was vibrated at 1 kHz under 20 V driving potential. Displayed values are the average amplitude measured at the center of each tile over three experiments. (C) Linear relationship between applied peak-to-peak driving potential and vibrational amplitude for frequencies of 0.5, 1, and 2 kHz. Points on graph are average amplitude with standard deviation measured at the center of the vibrating dish using laser vibrometry.

To nanovibrationally stimulate *E. coli* cells, we used an apparatus whose design has been adapted from that used for mammalian cell stimulation^19,25^ and which relies on reverse piezoelectricity to vertically vibrate cell suspensions contained in polystyrene Petri dishes (35 mm) (Figure 1A). Sample dishes were magnetically bonded to a metallic stage fixed on an aluminum plate. Four piezoelectrics were sandwiched between this and an aluminum base and were actuated through a signal generator and amplifier using sinusoidally oscillating voltages. The vibrational apparatus was located within an incubator which was kept at 30 °C to prevent any fluctuation in temperature from influencing experimental outcomes.

To homogeneously stimulate cells on surfaces, the vertical displacement needed to be uniform. We assessed this by partitioning the area of our vibrating stage into 144 equal-sized tiles and used a laser vibrometer to track the displacement at their center while vibrating at 1 kHz under 20 V driving potential. We found that tiles near the center of the stage vibrated in phase, at amplitudes of 40.0 ± 1.8 nm (Figure 1B) and used this location to load samples during vibrational experiments. Because the surface vertical displacement in this location is uniform (Figure 1B), cells will experience the same stimulation magnitude, regardless of their position on it, preventing bidimensional diffusion on the surface from interfering with cells stimulation.

We validated the relation between vibrational amplitude and applied voltage using laser vibrometry to measure the amplitude of the sample oscillations. For peak-to-peak driving potentials of up to 40 V and tested frequencies of 0.5, 1, and 2 kHz, the measured nanometric amplitudes of the sample dish rose linearly with the applied potential (Figure 1C). Surface nanovibrations mechanically stimulate cells by subjecting them to accelerative, compressive forces originating from the piezoelectric expansion and driving the sample vertical displacement. These forces act perpendicular to the sample’s surface and are transferred, through the attached cells, to the liquid weighting above them. Because of the liquid opposing inertia, the cell membrane would come under a compressive force which is proportional to the weight of the hovering liquid. Using Newton’s second law, it is possible to estimate the peak force acting on surface attached cells as
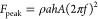
1where ρ is the density of the liquid (approximated to that of water at 30 °C), *a* is the area occupied by a cell on the surface, *h* is the height of the liquid suspension in the dish (6.0 mm), *A* is the amplitude and *f* the frequency of the vibrating surface.

Despite lacking the physical support of the surface, cells diffusing in suspension could also be subjected to nanovibrational stimulation. However, because this is poorly supported by experimental evidence, we estimated the peak magnitude of forces acting instead on surface approaching bacteria during adhesion using the same approach developed to interfere with mesenchymal cells osteogenesis in polystyrene dishes.^25−27^ However, contrary to mammalian cells, the surface orientation of rod-shaped Gram-negative bacteria such as *E. coli* during adhesion can vary from parallel to perpendicular to the surface, leading to different values of *a* and subsequent force estimates. To account for this variability, we quantified the average area occupied by cells on polystyrene dishes using image analysis on fluorescence pictures of surface attached *E. coli* cells after 2 h (Figure S1). The resulting value of 4.0 ± 0.9 μm^2^ was used together with measured vibrational amplitudes from applied potential of up to 40 V, to estimate the mean peak intensity of accelerative forces acting on cells on surfaces when these are vibrating at 0.5, 1, and 2 kHz (Figure S3).

These forces are of piconewton magnitudes applied to the whole cell and increase linearly with the applied potential. Because of the technical difficulty of their experimental determination, vibrational forces are commonly estimated through the above approach.^20^ In fact, while AFM can determine forces acting on a cell sitting on a vibrating piezoelectric, this would make for a different experimental setup and inconsistent force determination. Consequently, we followed the same approach validated in mammalian studies and estimated the forces acting on surface attached cells.^25−27^

The reason we chose piconewton forces was 2-fold: first, previous studies have shown that bacterial mechanosensors, such as type IV pili and *E. coli* flagellar motors, exert forces of similar magnitude during surface adhesion.^28−31^ Second, whereas ligand–receptor interactions and bacterial adhesion can reach nanonewton magnitudes,^32−34^*E. coli* K-12 adhesion on abiotic surfaces is not receptor mediated as it relies on amyloid fibers known as curli. For similar reasons, kilohertz frequencies were selected as they allow one to apply peak mechanical stimuli within milliseconds intervals. This is the time scale of flagella rotation^35^ and mechanosensitive channels opening,^36^ both force sensitive elements which partake surface sensing and adaptation^37^ and that could, therefore, be targeted by mechanical stimulation.

As a result, we believed nanovibrational stimuli of pN magnitude and kHz frequency to be suitable to influence natural adhesion and sensing of surface approaching bacteria.

### Nanovibrational Stimulation of pN Intensity Mitigates Bacterial Surface Adhesion

Using our vibrational apparatus, we investigated the influence that nanovibrational stimulation has on *E. coli* K-12 surface adhesion. A summary of this process and biofilm formation is provided in the Supporting Information. To minimize any damping effect that a conditioning layer could have on transferring mechanical stimuli from the surface, we washed and resuspended cells in minimal media before exposing them to pristine sterile polystyrene surfaces in Petri dishes. This was to reduce the number of surface impurities and to prevent any extracellular polymeric substances or cell debris from being carried from the culture to the sample, where they could form a conditioning layer.

To investigate the effect that mechanical stimulation has on surface adhesion, we vibrated the dishes containing cell suspensions for 2 h at 1 and 2 kHz and under driving potentials generating peak forces between 15 and 100 pN. We quantified the effect this had on cell adhesion by comparing the surface coverage between vibrated samples and controls (incubated under the same conditions without vibration) using fluorescence microscopy. To ease cells counting and quantification, we employed *E. coli* K-12 SCC1, a strain constitutively expressing GFP (Supporting Information Materials and Methods).

**Figure 2:**
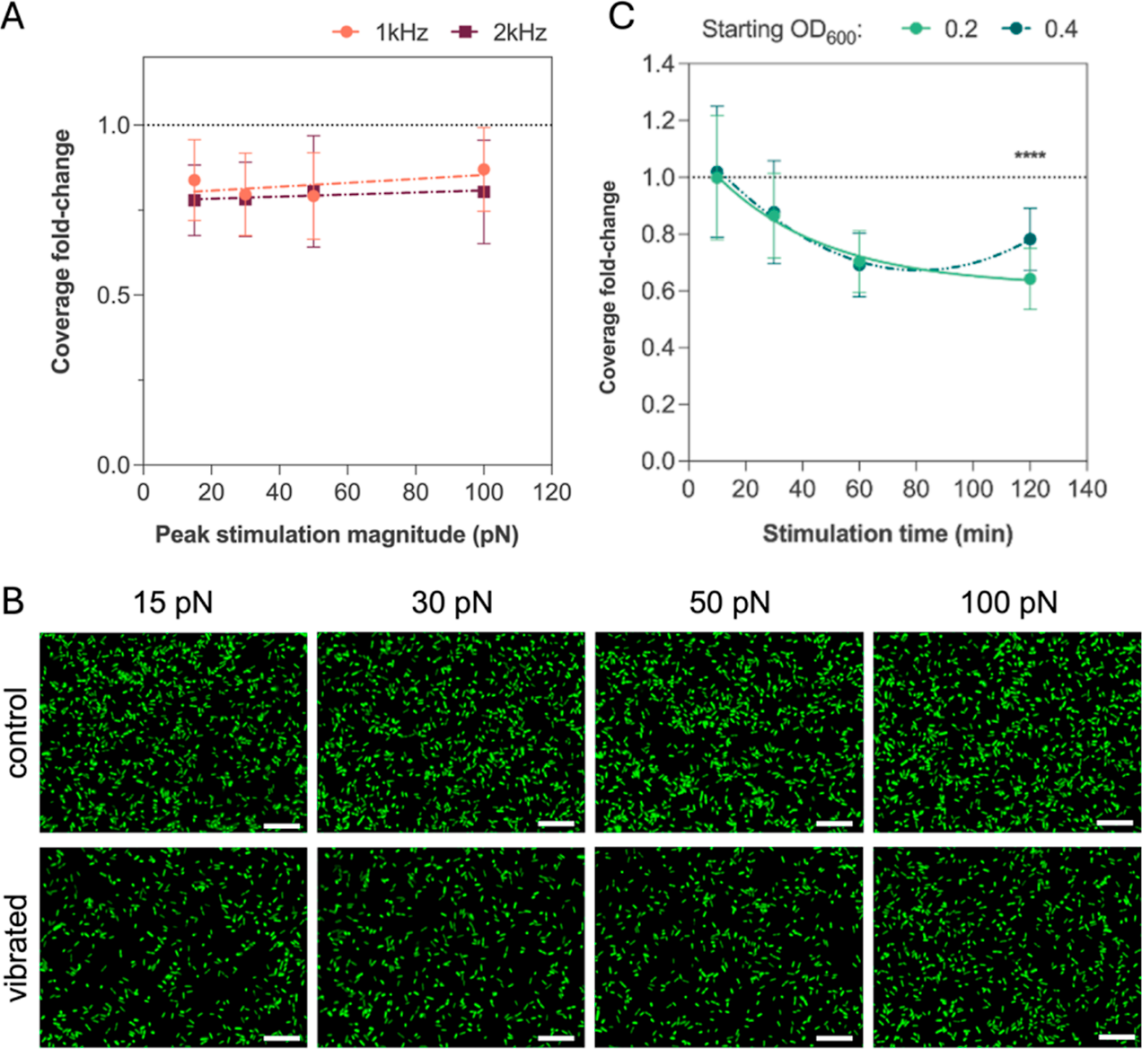
(A) Fold change in coverage of *E. coli* SCC1 cells on vibrated samples following stimulation between 15 and 100 pN at 1 and 2 kHz. Applied driving potentials to achieve each magnitude were derived from Figures 1C and S3. (B) Fluorescence images of cells on control and vibrated surfaces after stimulation at tested peak magnitudes. Scale bars are 15 μm. (C) Change in surface coverage over time following nanovibrational stimulation at 30 pN (2 kHz, 3.7 V) for starting OD_600_ of 0.2 and 0.4. Data on graphs represents the mean with standard deviation of the fold change in surface coverage in fluorescence images between control and vibrated samples (*n* > 121 per condition, ****p < 0.0001).

Following these experiments, we observed that mechanical stimulation reduced the surface coverage on all tested magnitudes and frequencies (Figure 2A,B). The effect on cells was independent of stimulation magnitude as adhesion decreased on average by 19 and 21% at 1 and 2 kHz, respectively. When decreasing the vibrational frequency to 500 Hz, we observed that the effect of vibrational stimulation on surface adhesion decreased for both tested magnitudes of 15 and 30 pN (Figure S4A). This suggests that while nanovibrational stimulation hinders surface adhesion, its effect on cells mostly depends on the frequency rather than the intensity of the applied stimuli.

To better understand nanovibrations’ influence on adhesion, we followed the change in surface coverage over time and observed that this depends on the length of the stimulation period. For nanovibrational stimulation of 30 pN peak magnitude (2 kHz, 3.7 V), we detected a statistically significant change in cell adhesion after 30 min which peaked at 1 h and diminished afterward, leading to the previously recorded 21% reduction after 2 h stimulation (Figure 2C, dark-green line). As cells grew in the samples during vibrational stimulation (Figure S5A), this resulted in increased cells surface sedimentation and clustering (Figure S5B). Specifically, we derived the fraction of cell sediments (Figure S2) and observed that this increased sigmoidally at a rate proportional to the starting suspension density (Figure S5C). Since clustered cells have a different mechanical sensitivity than homogeneously dispersed ones, cell concentration in the sample and subsequent sedimentation decrease the effect that vibrations have on adhesion, which is almost entirely suppressed for suspension densities beyond OD_600_ of 0.8 (Figure S5D). Mindful of these results, we halved cell concentration in the samples from 0.4 to 0.2 OD_600_ and observed that the fold change in surface coverage no longer diminished after 2 h (as in the 0.4 OD_600_ sample), but rather grew to 36% (Figure 2C, light-green line). This supports the idea that bacterial suspension density and surface sedimentation hinder nanovibrational stimulation and its effect on bacterial adhesion.

In our experiments, vibrational stimulation had been applied from the onset of bacterial surface adhesion. However, this is known to increase in strength the more time cells spend on the surface,^38,39^ as they transition to a sessile phenotype and become better equipped to hold grip on it (Figure S4C). We tested the effect this had on nanovibrational stimulation by allowing cells to colonize surfaces undisturbed from 15 min to 1 h, after which these were vibrated for 2 h at 30 pN (2 kHz 3.7 V) and we quantified the resulting change in surface coverage between samples and controls. Contrary to our expectations, the fold change in surface coverage between vibrated samples and controls was independent of cells colonization time (Figure S4D). Despite improving cells adhesion through surface precolonization, vibrational stimulation retained the same efficacy as when applied to fresh colonizers, suggesting its effect on cells adhesion and sessile transition is reversible.

Together, the above results show that mechanical stimulation of the piconewton intensity decreases *E. coli* surface adhesion. Under conditions accounting for passive sedimentation, the efficacy of nanovibrational stimulation on adhesion increases with stimulation time (Figure 2C), it is reversible (Figure S4D) and independent of the magnitude of the applied stimuli, even when this was increased up to 500 pN (2 kHz, 54.3 V, Figure S4B).

The way nanovibrations could bring about these effects is by either disrupting cells physiology or their physicochemical interactions with the surface. We found that nanovibrational stimulation had no influence on the adhesion of negatively charged polystyrene beads (Figure S7A) and was less effective on dead *E. coli* cells (Figure S7B). Moreover, using propidium iodide (PI) staining, we observed that nanovibrations did not permeabilize cell membranes (Figure S6), suggesting they do not damage cells envelope. This revealed that rather than interfering with cells passive physical interactions with the surface (i.e., hydrophobic, van der Waals and electrostatics) or damaging cells envelope, nanovibrations hinder adhesion by acting on cells’ physiology and surface behavior.

### Nanovibrational Stimulation Does Not Affect Cell Surface Motility

Flagellar motility and chemotaxis are known to promote surface adhesion and colonization in *E. coli*.^40^ Therefore, nanovibrational stimulation could decrease surface adhesion by altering cell motility. We tested this by stimulating cells at 50 pN peak magnitude (2 kHz, 5.4 V) and, after 2 h, we recorded 20 s videos of cells on the surface.

**Figure 3:**
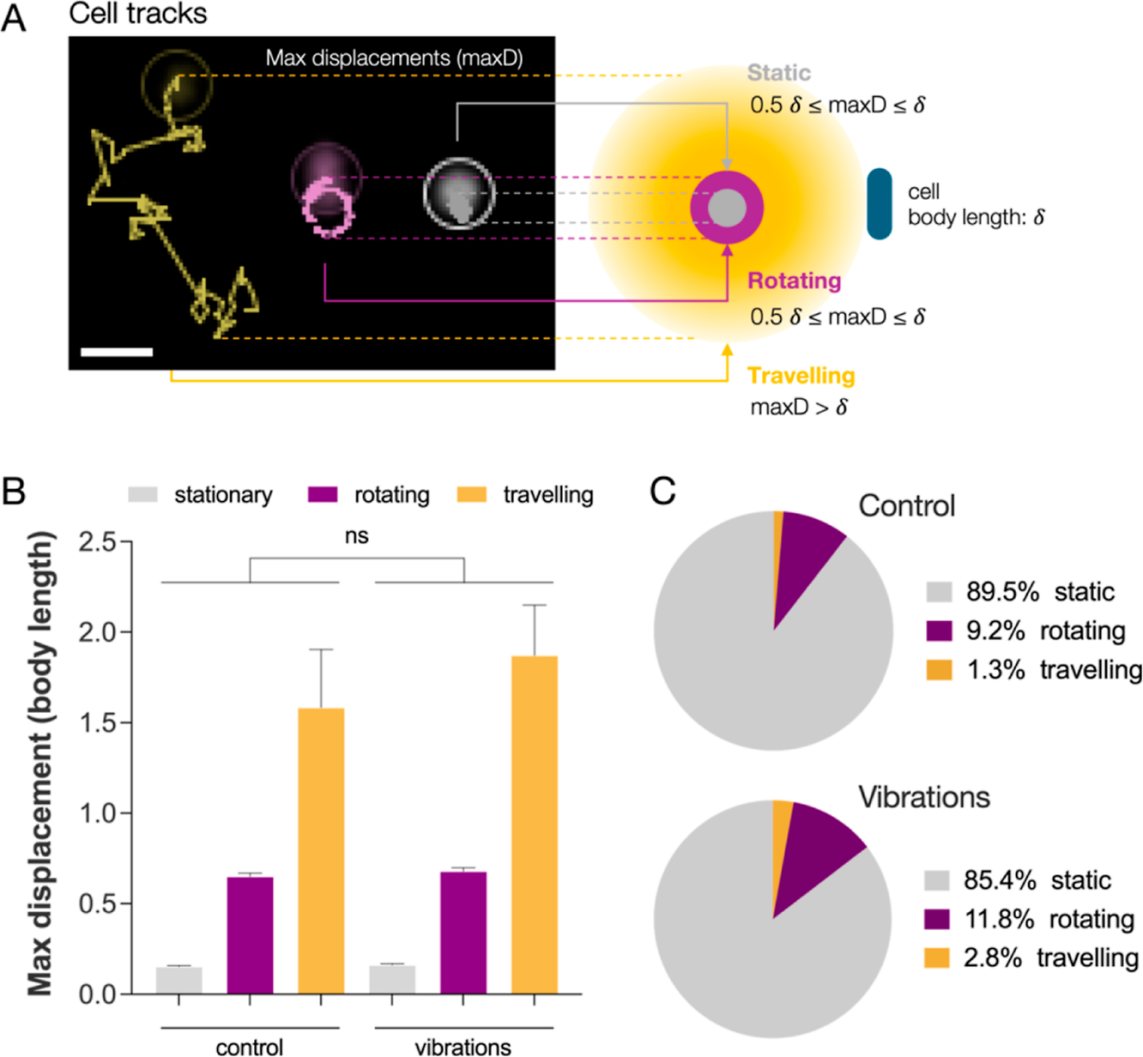
(A) Tracks from recorded videos of cells on surface and their classification into *static* (gray), *rotating* (purple), and *traveling* (yellow). Labels were assigned based on cells’ max displacement (maxD) relative to their measured average body length (δ). (B) Comparison of track’s max displacement for cells in each category between vibrated samples and control. (C) Total fraction of static, rotating, and traveling cells resulting from tracks analysis and their classification after 2 h on controls and vibrated samples (*n* = 1422 and 1748 for control and vibrations respectively, scale bar 2 μm).

We applied tracking algorithms^41,42^ on the recorded videos, and from the resulting cell-specific trajectories, we derived cells’ maximum displacement (maxD) and mean body length (δ), which we used to classify cells as either stationary (maxD < 0.5 δ), rotating (0.5 δ < maxD < 1 δ) or traveling (maxD > 1 δ) (Figure 3A). We confirmed the reliability of our methodology by applying it to conditions where a difference in motility was expected such as fast-growing cells in LB, slow-growing ones in M63+, and abiotic particles. As anticipated, all three conditions had distinct motility profiles with fast growing cells and abiotic particles, respectively, moving the most and the least on the surface (Figures S8 and S9).

Following video analysis, we did not observe any statistically significant difference in the average max displacement of static, rotating, and traveling cells upon vibrational stimulation (Figure 3B). Moreover, the fraction of cells in each category changed little between vibrated samples and controls (Figure 3C). In fact, most of the tracked cells lay stationary on the surface as only 14.6% and 10.5% were either rotating or traveling in vibrated and control samples, respectively. Our findings show that after 2 h, nanovibrational stimulation does not cause any significant difference in surface motility. Nanovibrational stimulation is, therefore, to affect another side of the cell’s physiology with negative consequences on adhesion. Mechanical sensing is central to bacterial adhesion, and nanovibrational cues share the same magnitude as those generated and sensed by bacteria during surface attachment. We, therefore, investigated how nanovibrational cues might interfere with cell’s surface sensing.

### Membrane Potential Is Necessary for Nanovibrational Stimulation to Hinder Surface Adhesion

For nanovibrational stimulation to influence surface sensing, this needs to act on mechanosensory elements whose signaling cascade leads to surface adhesion. While no such mechanosensing pathway has yet been identified in *E. coli*, mechanical stimuli have been reported to alter cell membrane potential with an effect on protein concentration.^43^ Moreover, membrane potential has also proved fundamental for cells sessile transition on surfaces.^44^ As a result, we investigated if membrane voltage is necessary for cells’ response to nanovibrational stimulation.

**Figure 4:**
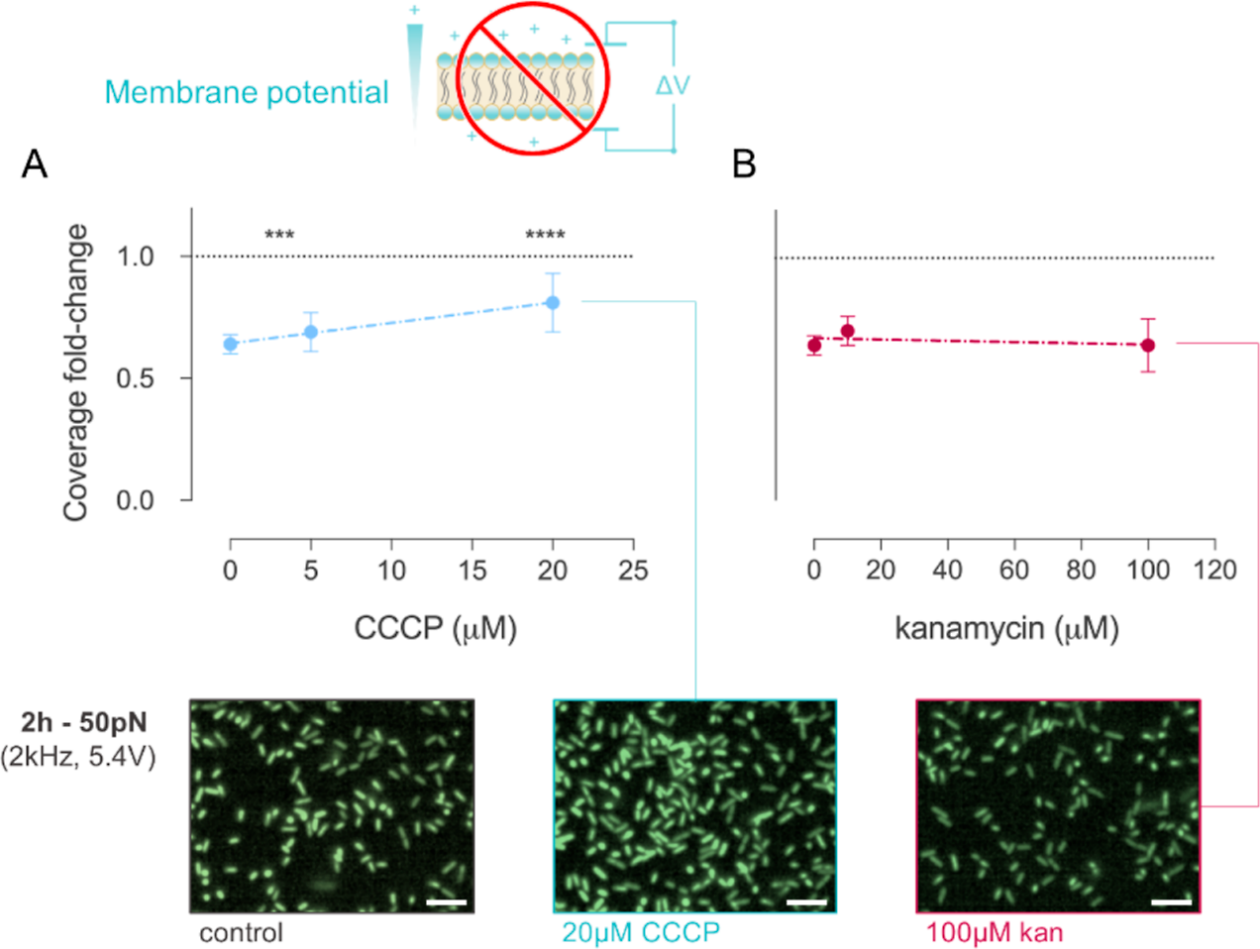
Change in surface coverage following 2 h stimulation at 50 pN (2 kHz, 5.4 V) for cells treated with different concentrations of the membrane protonophore CCCP (A) and the ribosome targeting antibiotic kanamycin (B). Data represent the mean with SD of the fold change in coverage between vibrated samples and controls (*n* > 105 for all conditions, ****p < 0.0001, scale bar 2 μm).

We assessed this by progressively dissipating cell membrane potential with the membrane protonophore carbonyl cyanide 3-chlorophenylhydrazone (CCCP, Figure S10) and compared the change in surface coverage between stimulated and control samples. We observed that the fold-change in coverage between vibrated samples and controls decreased with increasing CCCP concentration, almost halving the effect on cell adhesion at 20 μM CCCP (Figure 4A). To exclude that these findings were the result of a cytotoxic effect of CCCP, we repeated the experiments by treating cells for 2 h with the ribosome targeting kanamycin and observed that this has instead no influence on nanovibrational stimulation (Figure 4B).

The above findings show that membrane potential is necessary for nanovibrational stimulation to influence cell adhesion, as mechanical stimulation is less effective on membrane depolarized cells. Furthermore, cell rapid response stimulation coupled to their retained sensitivity upon kanamycin treatment after 2 h revealed that a major part of cells response to nanovibration does not involve gene expression and protein translation.

### Nanovibrational Stimulation Alters the Polarization of Cell Membrane Potential

Membrane potential dynamics have been observed to influence several physiological processes in bacteria^45−47^ and bioelectric signal transduction appears to be a common feature in both Gram positive and negative species.^48^ Therefore, membrane voltage could transduce mechanical stimuli and partake to the signaling cascade, which lead to cell adhesion. As a result, nanovibrational stimulation could hinder surface adhesion by disrupting cell membrane voltage.

We investigated the effect of nanovibrational stimulation on cell membrane potential by stimulating our samples for 2 h at 50 pN peak magnitude (2 kHz, 5.4 V), and monitored changes in cell membrane voltage of surface attached cells using the fluorescent probe 3,3′-diethyloxacarbocyanine iodide [DiOC_2_(3)]. This is a positively charged polarization sensitive dye of known Nernstian behavior in *E. coli*.^49^ Its chemical structure makes it shift from green to red fluorescence as it electromigrates in membrane polarized cells. While this can be used to determine the fraction of polarized cells by measuring the ratio of green (total) to red (polarized) fluorescing cells, direct measurement of the dye fluorescence intensity at 670 nm can reveal the extent of membrane polarization in *E. coli*.^49^

**Figure 5:**
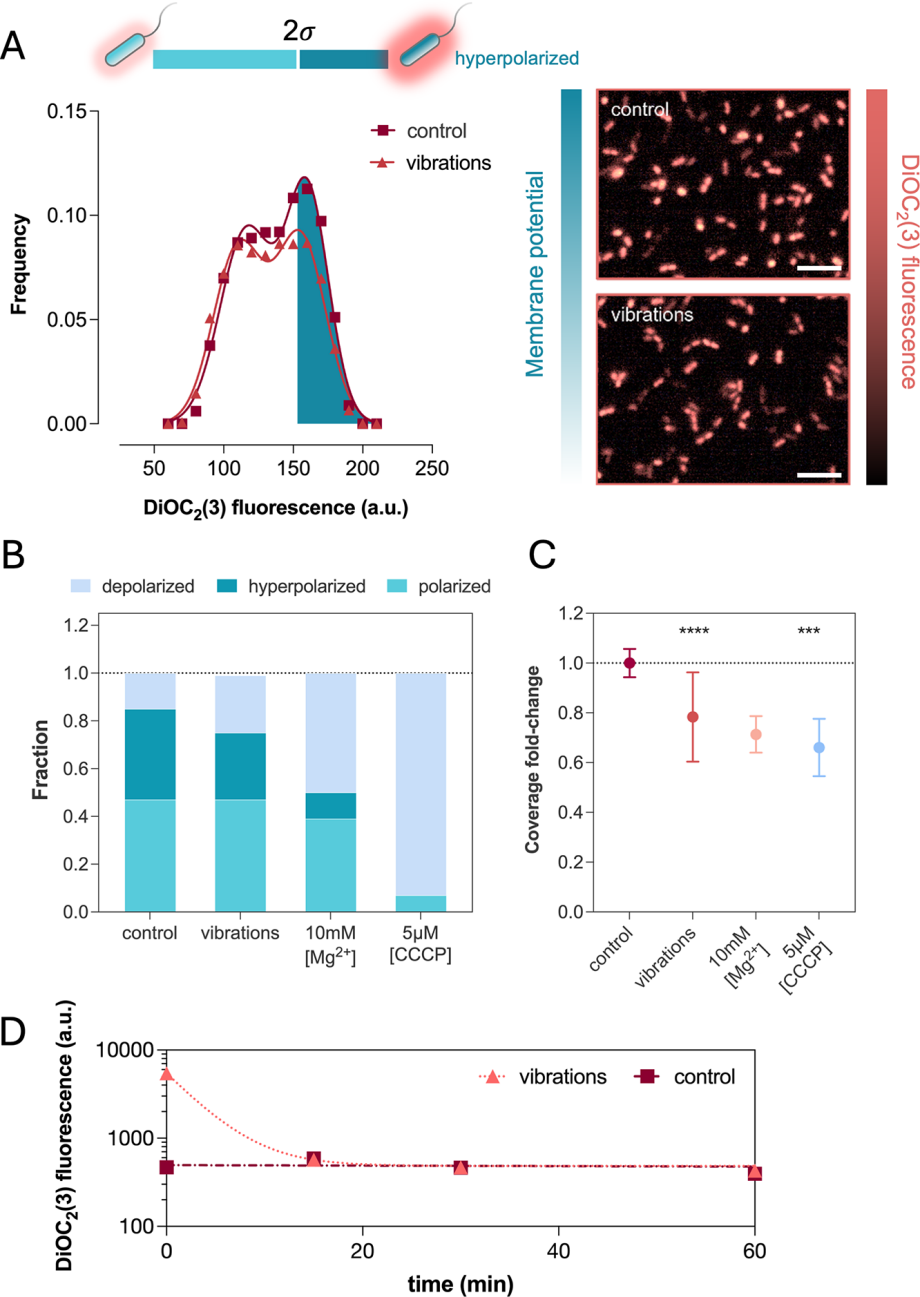
(A) Frequency distributions of DiOC_2_(3) fluorescence at 670 nm of cells on surfaces of control and vibrated samples at 50 pN (2 kHz, 5.4 V). Shaded areas are the fraction of hyperpolarized cells as integrals under the Gaussian interpolating curves taken beyond 2 standard deviations (σ) from the leftmost peak in control samples (# imaged cells >1.6 × 10^4^, *R*^2^ > 0.98 for both conditions). Inserts are images of cells fluorescence on the surface of control and vibrated samples (scale bars are 10 μm). (B) Fractions of cells polarization on surfaces for different conditions. (C) Effect of dissipating membrane potential on surface adhesion following mechanical stimulation (50 pN, 2 kHz, 5.4 V) and chemical treatment with 10 mM Mg^2+^ or 5 μM CCCP (*n* > 112 per condition, data on graph are the mean with SD of the fold change in coverage of fluorescence pictures between samples and controls, ****p < 0.0001). (D) Flow cytometry data of DiOC_2_(3) fluorescence at 670 nm of planktonic cells over 2 h after vibrational stimulation. Lines are exponential and linear interpolation curves, (*R*^2^ > 0.96).

To prevent differences in surface coverage caused by vibrational stimulation from biasing our results, we stained cells and did not wash the sample surfaces prior to microscopy. While the number of imaged cells was the same on both vibrated samples and control (2.2 and 2.3 × 10^4^, respectively), DiOC_2_(3)-red emission from stained cells on the surface was bimodally distributed as a polarized population that coexists with a hyperpolarized one centered at +2σ (Figure 5A).

From ratio-metric measurements, vibrated samples and controls shared the same fraction of polarized (47%), but not depolarized cells (15 and 25%, respectively, Figure 5B). On vibrated samples, the increase of depolarized cells was met with an equal decrease in hyperpolarized ones (from 38 to 28%) suggesting that after 2 h nanovibrational stimulation reduces cells’ hyperpolarization on the surface.

To test whether the lack of membrane hyperpolarization could connect to cells’ decreased adhesion on vibrated samples, we suppressed cells hyperpolarization by increasing the ionic strength of the medium using MgSO_4_. Magnesium ions has been shown to prevent membrane hyperpolarization by hindering cation effluxes^50,51^ and we observed that treating cells with 10 mM MgSO_4_ had a similar effect under our conditions, decreasing the number of hyperpolarized cells from 38 to 11% (Figures 5B and S11). To prevent differences in motility and sedimentation speeds caused by the increase in ionic strength from affecting our results, we adjusted the cells’ suspension density to lead to the same surface coverage after 2 h, before washing the samples and imaging the surface (Figure S12). We observed that treating cells for 2 h with 10 mM MgSO_4_ decreased cells surface coverage relative to untreated controls (Figure 5C, pink), while complete depolarization with 5 μM CCCP further decreased adhesion only by a small margin (Figure 5C, light blue). This suggests that membrane hyperpolarization is a relevant factor to cells’ natural surface adhesion and that either chemical or mechanical disruption would hinder the process (Figure 5B,C).

This concomitant presence of diminished surface hyperpolarization and reduced adhesion on vibrated samples could indicate the impaired attachment of more highly polarized cells, which would accumulate in suspension. We, therefore, monitored the membrane potential of planktonic cells immediately after vibrational stimulation (2 h, 2 kHz, 1 V), and up to 2 h afterward by staining cells with DiOC_2_(3) and by analyzing the samples liquid suspension through flow cytometry. Immediately following vibrational stimulation, planktonic cells displayed a 10-fold increase in fluorescence intensity relative to nonvibrated controls (Figure 5D). This fluorescence rapidly decreased through an exponential decay, regressing to the same level as control cells within 30 min after stimulation. This revealed that cells in suspension have an average membrane potential that is more polarized during nanovibrational stimulation.

Together, the above results support the hypothesis that nanovibrational stimulation mitigates surface adhesion by targeting cell membrane potential, depolarizing that of cells on the surface and ultimately interfering with the adhesion dynamic of more highly polarized cells.

## Conclusions

Within this work, we tackled the question of whether mechanical stimulation in the form of nanometric surface vibrations could hinder bacterial adhesion. Our findings demonstrate that, over 2 h, nanovibrational stimulation of kilohertz frequency and piconewton peak magnitude consistently mitigates surface adhesion in *E. coli* K-12.

Under ideal conditions, mechanical stimulation decreased cell coverage on polystyrene surfaces by up to 36% (Figure 2C). As nanovibrational stimulation had little to no effect on abiotic particles or dead cells (Figure S7), did not damage cells envelope (Figure S6), and had no effect on surface motility (Figure 3B,C), we conclude that nanometric surface vibrations mitigate adhesion, not by disrupting physicochemical interactions, but by acting instead on cells’ physiology and sensing. Moreover, since nanovibrations continued to affect cell surface adhesion over 2 h when these were treated with the translation inhibitor kanamycin, this suggests that cells response to vibrations is predominantly independent from de novo protein expression.

**Figure 6:**
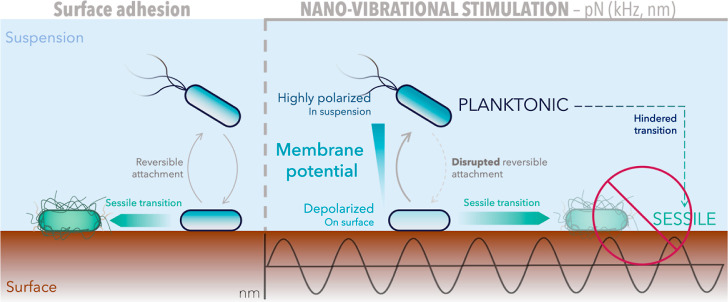
Effect of nanovibrational stimulation on *E. coli* surface adhesion and sessile transition. During surface colonization, planktonic cells in suspension (left) reversibly attach to the surface and subsequently transition to a sessile, surface associated phenotype leading to biofilm formation. Nanovibrational stimulation of piconewton intensity (right) targets cell membrane potential, depolarizing cells on surface and hindering the attachment of highly polarized ones. This disrupts cells reversible attachment dynamics, so reducing surface adhesion, sessile transition, and biofilm formation.

In fact, for this to occur, cells need a dynamic membrane potential (Figure 4A) whose polarization is disrupted by nanovibrational stimulation as this increases the fraction of depolarized cells while reducing that of hyperpolarized ones on the surface, so disrupting their reversible adhesion and causing their accumulation in suspension (Figure 5A,B,D). Preventing membrane hyperpolarization by treating cells with higher concentrations of Mg^2+^ similarly impaired surface adhesion (Figure 5C) suggesting that membrane hyperpolarization supersedes the adhesion and sessile transition of surface attached cells. Combined, our findings reveal that nano vibrational stimulation targets cell membrane potential, altering its polarization and thus interfering with its complex regulation of surface adhesion and subsequent transition from planktonic to sessile lifestyle (Figure 6).

Bioenergetics have previously been reported to be involved in bacterial motile-sessile switch. Membrane potential has also been observed necessary to *Vibrio cholerae*(44) sessile transition while *E. coli* has been shown to display a decrease in respiratory rate upon surface attachment.^52^ Moreover, a decrease in membrane potential has been reported to reduce motility and increase attachment in *Salmonella typhimurium*.^53^ While the mechanisms underpinning these responses are currently unknown, it appears that membrane potential plays a pivotal role in the bacterial motile-sessile switch. Therefore, by interfering with the polarization of cell membrane potential, nanovibrational cues could prevent downstream signaling events leading to adhesion and sessile transition.

Considering recent studies investigating the intracellular events occurring when *E. coli* approaches a glass surface,^15^ we propose that flagella could act as the primary vibrations’ mechanosensors. Their motor could mediate the transduction of a mechanical signal into the observed change in membrane potential which could subsequently alter the intracellular concentration of c-di-GMP.^15^ Intracellular c-di-GMP has previously been shown to allosterically control synthesis of the exopolysaccharide poly-*N*-acetylglucosamine in *E. coli* that could thereby alter surface adhesion.^54^ To simultaneously monitor flagella biosensing capabilities, we plan on using single motor bead assays based on tethering cells flagella to either AFM tips or abiotic beads which are then optically entrapped.^55^ Alternatively, we suggest that nanovibrational stimulation could cause the uncontrolled opening of mechanosensitive or ion channels, so altering the delicate intracellular pH balance that links to c-di-GMP signaling following surface contact in *E. coli*.^15^ Whatever the mechanism, we would expect that sensing and response to vibrations would be influenced by other environmental factors and physicochemical cues important in surface sensing such as osmolarity.^37^ Although we have shown that short-term response to vibration does not require de novo protein synthesis and thereby is gene-expression-independent, RNA sequencing of vibrationally stimulated cells could be used to investigate if gene expression plays a role in longer-term responses to vibration.

While the observed increase in the membrane potential of planktonic cells is consistent with disrupted attachment dynamics of highly polarized cells, it is tempting to think that cells in suspension might also be sensitive to vibrational stimulation through a yet unknown mechanism that we set to investigate in future studies.

A crucial question regarding potential applications of vibration as an antibiofilm strategy is its applicability to other bacterial species; this is the subject of future work. However, given the relatively widespread distribution of mechanosensing mechanisms in bacteria, we would expect that this phenomenon is not restricted to *E. coli*. Indeed, *P. aeruginosa*, with two known mechanosensitive pathways (flagella and type IV pili), might be expected to be more sensitive to vibrational stimulation.

The observed mechanical disruption of cells membrane voltage could explain previous experimental efforts targeting biofilm development using vibrational cues where a similar effect could be at play.^22,24,56,57^ Mindful of this tickling sensitivity, biofilm control strategies could be enhanced by shifting attention from the surface to the cell. Suppressing surface adhesion has become a recurring countermeasure to biofilm formation, one that is mainly achieved through the modification of the surface physical and chemical properties,^58−61^ with usually short-term antibiofouling effects. Controlling bacterial behavior through mechanical stimulation could prove a technologically appealing alternative which would complement existing antibiofouling approaches, leading to multipronged strategies capable of exploiting both surface properties and cells physiology. Seeing the low cost and ample flexibility of vibrational approaches, such a future is in close experimental reach.

Finally, while we focused on surface adhesion, it is intriguing to think that nanovibrational stimulation could also mitigate other mechanically dependent behaviors such as virulence. As this heavily depends on mechanical cues in both *P. aeruginosa* and pathogenic *E. coli*,^62−65^ it represents a strategic target for nanovibrational control.

To conclude, our work shows how the use of nanometric surface vibrations mitigates bacterial adhesion on hard surfaces by disrupting cell membrane potential. Future work will explore the mechanism through which nanovibrations are bioelectrically transduced into altered membrane potential and reduced surface attachment. We believe that our findings could vouch for the possibilities that awaits when mechano-biology is exploited to control bacterial behavior, serving a prime opportunity to harness and learn from tickling bacteria.

## Experimental Section

### Vibrational Apparatus

The vibrational device was made of a 12 × 12 × 6 cm aluminum base (*L* × *W* × *H*) on top of which four piezoelectric actuators (PL088.30, Physik Instrumente, Karlsruhe, Germany) were connected in series and fixed using thermoresistant glue. Glued on top of these was a 12 × 12 × 0.6 cm aluminum plate (*L* × *W* × *H*) covered with a steel sheet (1 mm thick) which served a stage for our samples. These consisted of 35 mm Petri dishes (Starsted) containing 5 mL of bacterial suspension and carrying a 1 mm thick steel disk fixed at their outer bottom with epoxy glue (Loctite, Hempstead UK). A neodymium magnet (Magnet Store Ltd., Wigan UK) was then placed at the stage’s center and used to bind a single sample dish per experiment. We found that any magnetic interference on surface adhesion was negligible under our conditions as we did not observe any significant change in surface coverage on samples loaded on the magnet (Figure S13). The apparatus was situated in an incubator at 30 °C. To induce vibrations and power the piezo array, we used an amplifier (PD200, PiezoDrive, NSW, Australia) that magnified 20 times the sinusoidally oscillating potential sent from a signal generator (Tektronix AFG3022C, USA).

### Laser Vibrometry Characterization

We used laser vibrometry (laser unit OFV 534 and controller OFV-5000, Polytec, Germany) to assess the vibrational amplitudes of the stage and sample dishes for a range of applied sinusoidal voltage at frequencies of 0.5, 1, and 2 kHz. For each frequency and driving potential, the mean value and standard deviation of the measured amplitudes were used to calculate the peak magnitude of the stimulation force (eq 1).

### Bacterial Strains and Media

On all experiments, we used either *E. coli* MG1655 or its SCC1 derivative.^66^ Cells were grown in M63+ minimal medium overnight and then resuspended in 5 mL of fresh medium with polystyrene sample dishes (35 mm diameter) at desired OD_600_.

### Fluorescent Microscopy

Surface imaging of sample dishes was performed using a Zeiss Axiolab E-re (mercury lamp: Osram HBO 50 W AC L1, camera: Moticam 1080 Motic Scientific, objective: Zeiss Achroplan 40*×*/0.8 numerical aperture Ph2 water immersion, filters: Zeiss Axio Cube Filter Slider 4 FL 446425, Carl Zeiss 488050-8003 BP 690/50, 46409 D460/50 M and 30217 D540/40 M).

### Cell Size Quantification

*E. coli* SCC1 cells from 0.2 OD_600_ suspensions in M63+ were left to attach to the bottom surface of 35 mm Petri dishes (Starsted) for 2 h. Samples were then rinsed twice by placing them into plastic boxes containing 400 mL of PBS which were mechanically shaken for 90 s. To hold samples in position, magnets were fixed at the boxes’ outer bottom. Finally, 30 to 40 pictures of the surfaces were gathered per sample using fluorescence microscopy. These were digitally processed, and the average surface area occupied by the cells was calculated from three replicates (Supporting Information Materials and Methods).

### Vibrational Stimulation

*E. coli* SCC1 suspensions at either OD_600_ of 0.2 or 0.4 within polystyrene Petri dishes were vibrated for 2 h at intensities between 15 to 500 pN and frequencies of 0.5, 1, and 2 kHz under applied driving potentials derived from Figures 1C and S3. After stimulation, samples and controls were rinsed twice by placing them into plastic boxes containing 400 mL of PBS which were mechanically shaken for 90 s. To hold samples in position during this step, magnets were fixed at the box’s outer bottom. Finally, 30 to 40 pictures of the surfaces were gathered per sample using fluorescence microscopy. These were digitally processed and the relative change in coverage across three replicates was expressed as the average coverage from vibrated samples normalized by the mean of the associated control (Supporting Information Materials and Methods).

### Over Time Vibrational Stimulation

*E. coli* SCC1 suspensions from overnight cultures in M63+ were prepared at both OD_600_ of 0.2 and 0.4 within polystyrene sample dishes and subsequently vibrated at 30 pN peak magnitude (2 kHz, 3.7 V) for either 10, 30, 60, or 120 min. Samples were then rinsed, and the change in surface coverage was quantified as explained above.

### Vibrational Response of Antibiotic Treated Cells

*E. coli* SCC1 suspensions in sample dishes (OD_600_ of 0.2) were supplemented with concentrations of kanamycin (10 and 100 μM, Thermo Fisher) or CCCP (5 and 20 μM, Fisher Scientific) and subsequently stimulated for 2 h at 50 pN (2 kHz, 5.4 V). Samples were then rinsed and imaged, and changes in surface coverage were determined as described above. All relevant experiments were performed in triplicate.

### Changes in Cells Polarization Following Vibrational Stimulation and Magnesium Treatment

*E. coli* MG1655 suspensions in sample dishes (OD_600_ of 0.05) were stimulated for 2 h at 50 pN (2 kHz, 5.4 V). During the final 20 min, these were stained using 150 μM DiOC_2_(3) (Thermo Fisher) and their membrane permeabilized by adding 11 mM EDTA. To this end, 4 mL of samples suspension were replaced with the same volume of a staining mixture in M63+ containing dye and EDTA amounts required to reach the above working concentration. To prevent accidental damage to the sample and device while performing this step, vibrational stimulation was suspended. After staining, vibrational stimulation was ceased, and samples were diluted 1 in 25 by twice replacing 4 mL of stained suspension with the same amount of fresh M63+. Up to 20 pictures were gathered from the surface as pairs on both the green and red channels from which we derived the fraction of polarized cells (Supporting Information Materials and Methods). All of the relevant experiments were performed in triplicate. When testing magnesium effect on membrane potential, its concentration was set to 10 mM using MgSO_4_ (Thermo Fisher). Samples were then incubated for 2 h and then processed and imaged as above for vibrated samples.

### Flow Cytometry Analysis of Cells Membrane Polarization in Suspension

*E. coli* MG1655 suspensions in sample dishes (OD_600_ of 0.2) were stimulated for 2 h (2 kHz, 1 V). During the final 20 min, these were stained using 150 μM DiOC_2_(3) (Thermo Fisher) and their membrane permeabilized by adding 11 mM EDTA. Aliquots (1 mL) were taken immediately after stimulation and up to 2 h afterward from vibrated samples and nonvibrated controls, centrifuged for 5 min at 3200*g*, resuspended in filtered PBS to an OD_600_ of 0.1, and analyzed using flow cytometry using a BD Accuri C6Plus instrument. Cells were illuminated with a 488 nm laser and fluorescence detected using 530/30 and 670LP filters.

### Changes in Surface Colonization Following Magnesium and CCCP Treatment

*E. coli* SCC1 cultures in M63+ were resuspended in sample dishes at OD_600_ of 0.2. These were supplemented with either 10 mM MgSO_4_ or 5 μM CCCP and then incubated at 30 °C for 2 h. Control samples were supplied with 1 mM Mg^2+^ and no CCCP. Between 30 and 40 surface pictures were gathered per sample and the relative change in coverage across three replicates was determined as the samples average normalized by the mean of associated controls (Supporting Information Materials and Methods).

### Vibrations Effect on Cells Motility

*E. coli* SCC1 suspensions from overnight cultures were resuspended in sample dishes (OD_600_ of 0.02) and vibrated for 2 h at 30 pN (2 kHz, 3.7 V). Swimming cells and supernatants were removed by replacing 4 mL of suspension with an equal volume of motility buffer (0.2% d-glucose in PBS) and three to four videos (20 s, 30 fps, Moticam 1080, Motic Scientific) of cells on surfaces were recorded at random locations. The above experiments were performed in triplicates and the resulting 9 to 12 videos per conditions were analyzed using FIJI’s plugin TrackMate^41,42^ (Supporting Information Materials and Methods).

### Data Analysis and Statistical Comparison

To statistically analyze samples, both parametric and nonparametric tests were used depending on data normality and homoscedasticity. When both conditions were met, we used either *t* tests or ANOVA, when only the latter was met, Welch’s correction to the preceding techniques was used instead. Finally, the Mann Witney *U* test and Kruskal–Wallis test were employed when samples were neither normally distributed nor homoscedastic. All statistical analyses were performed in R and GraphPad Prism while data handling was performed by using Microsoft Excel and RStudio.

## Supporting Information Available

Bacterial strains; media preparation; surface sedimentation; dead cells; abiotic particles; PI staining; CCCP depolarization and image processing; and supporting figures (PDF)
