# Supplementary material for: Nanovibrational Stimulation of Escherichia coli Mitigates Surface Adhesion by Altering Cell Membrane Potential
Source: ACS Nano. 2024 Oct 22;18(44):30786–97. doi: 10.1021/acsnano.4c11000 (PMC11544934; doi:10.1021/acsnano.4c11000)
Supplement: Supplementary file 1 — nn4c11000_si_001.pdf [file nn4c11000_si_001.pdf]

Supporting Information for

Nano-vibrational stimulation of *Escherichia coli* mitigates surface adhesion by altering cells membrane potential.

Dario G. Bazzoli, Nasim Mahmoodi, Terri-Anne Verrill, Tim W. Overton* and Paula M. Mendes*

[a] School of Chemical Engineering, University of Birmingham, Birmingham, B15 2TT, UK

* to whom correspondence may be addressed:

Prof. Paula M. Mendes – Email: p.m.mendes@bham.ac.uk

Dr Tim W. Overton - Email: t.w.overton@bham.ac.uk

Summary of *E. coli* K-12 adhesion and biofilm formation

E. coli K-12 is a widely used model organism and its adhesion and biofilm formation has been widely studied.[1] Primary adhesion to abiotic surfaces is thought to be mainly mediated by curli, short (0.5 - 1 µm) amyloid fibres projecting from the bacterial surface.[2] Curli also mediate attachment to human cells via extracellular matrix proteins. Synthesis of curli is regulated at multiple levels. Expression of the structural curli operon, *csgBAC*, is controlled by >15 transcription factors that integrate multiple diverse environmental stimuli.[3] The master regulator of curli synthesis is the transcription factor CsgD. In addition, curli production is regulated by >5 small RNAs.[4] Two further *E. coli* protein adhesins are type 1 fimbrae (0.2 – 2 µm long fibres), which mediate adhesion to mammalian cells via mannose and are important in attachment to biotic surfaces,[5] and antigen 43, which mediates cell to cell adhesion.[6]

E. coli K-12 also synthesises two exopolysacchrides. Poly-β-1,6-N-acetyl-glucosamine (PNAG) is a linear polysaccharide whose synthesis is allosterically regulated by the second messenger c-di-GMP.[7] Colanic acid is a branched exopolysaccharide comprising glucose, galactose, fucose and glucuronic acid.[8] Colonic acid synthesis involves a 19-gene cluster whose regulation is relatively poorly understood; the Rcs system is a known activator, but the exact stimuli for this regulation are unknown.

Biofilm formation is a multi-step process. Bacterial initially approach a solid surface and reversibly attach, usually mediated by adhesins; in this case, curli are thought to be the adhesin for abiotic surfaces. Next, bacteria become irreversibly attached. The exact processes occurring from the reversible-irreversible transition are unknown, but could involve protein adhesins and polysaccharides. Once attached, bacteria acquire a surface associated sessile phenotype and start multiply and form microcolonies, which, as they

become larger and more structured, turns into biofilms. Adhesins and polysaccharides are again involved in this structuring process. Finally, bacteria can disperse from the biofilm.

In this study, we focus on attachment of bacteria to a solid surface, the first steps of biofilm formation.

Materials and Methods

Surface samples preparation. Samples were assembled from triple vented polystyrene sterile dishes (35 mm diameter, Sarstedt) to which a 34 x 1 mm (L x H) polished iron disk was fixed at the outer bottom surface using epoxy-glue (Loctite).

Bacteria and Media. We employed *E. coli* K-12 MG1655 and its derivative SCC1[9] which constitutively expresses GFPmut3. For each strain a single colony from an LB agar plate was collected with a sterile inoculation loop, inoculated within a 120 mL flask containing 10 mL of M63+ minimal medium and incubated overnight at 30 ˚C and 150 rpm. LB agar plates with colonies were kept at - 4 ˚C and employed when less than a week old. All strains came from 50 % glycerol stocks stored at - 80 ℃ and refreshed monthly. LB broth (Sigma-Aldrich) was prepared in deionised water, autoclaved before use, and stored at room temperature for up to two months. The low osmolarity and minimal medium M63+ was prepared as a variation of standard M63. This uses succinate as an extra carbon source and has reduced glucose content (10 mM, 0.2 %, w/v). 5 X stocks contain the following chemical composition: $(NH_4)_2SO_4$ (75 mM, Sigma-Aldrich), KH_2PO_4 (500 mM, Sigma-Aldrich), sodium succinate (85 mM, Thermo Fisher) and $FeSO_4$ (9 µM, Sigma-Aldrich). The mixture was then neutralised to pH 7 with KOH 5 M (Sigma-Aldrich). Before use, the medium was diluted to 1 X in deionised water and supplemented with $MgSO_4$ (1 mM, Thermo Fisher) and D-glucose (10 mM, 0.2 % w/v, Thermo Fisher). Stock solutions were stored at - 4℃ for up to six months.

Surface sedimentation fractions. *E. coli* SCC1 cultures were resuspended in sample dishes at an OD_{600} of 0.2, 0.4 or 0.8. For each condition samples were incubated at 30 ˚C for 1, 10, 30, 60, 120 and 240 minutes. After this, 10 to 15 fluorescence pictures of the surface were gathered per sample and analysed to determine the fraction of cells sediments as described below (*image processing*). The resulting average coming from three independent replicates was plotted and interpolated using sigmoidal curves for each time point and starting suspension density.

Vibrational response of polystyrene functionalised beads and dead cell. *(i) Abiotic particles.* Fluorescent carboxy and amine functionalised beads (L4655 and L2778, Sigma-Aldrich) were used from stock to prepare OD_{600} 0.6 suspensions in PBS within sample dishes which we vibrated for 2 hours at 30 pN (2

kHz, 3.7 V). Surfaces were then washed and imaged to determine beads coverage as discussed below (*image processing*). All experiments were performed in triplicate. *(ii) Dead cells.* Overnight *E. coli* SCC1 cultures in M63+ were harvested and resuspended in 15 mL of fresh medium within 50 mL falcon tubes at OD_{600} 0.4. Cells were then treated with kanamycin (50 µg/mL, 100 µM) for 24 hours at room temperature and cell death was confirmed by the lack of cell growth on LB agar plates. Dead cells were then resuspended in 5 mL of fresh M63+ at an OD of 0.4 then transferred into sample dishes and vibrated for 2 hours (30 pN, 2 kHz, 3.7 V). Samples were then washed and surface imaged to quantify cells coverage on samples and controls. Experiments were performed in independent triplicates.

PI staining and vibrational damage on cells membrane. Overnight *E. coli* SCC1 cultures in M63+ were resuspended within sample dishes at OD_{600} of 0.2 and vibrated for 2 hours at 30 pN (2 kHz, 3.7 V). During the last 20 minutes of stimulation, cells were stained with Propidium Iodide (18 µM working concentration, Thermo Fisher LIVE/DEAD Bac-light kit). This was done by replacing 1 mL of suspension with the same volume of a staining mixture in fresh M63+. To prepare this, PI was diluted in the ratio of 1 µL of stock per mL of sample. To prevent damage to the sample and device, vibrations were stopped during this step. After staining, vibrational stimulation was suspended, samples washed, and 30 fluorescence pictures of the surface were gathered on both the vibrated sample and its control. These were then analysed to derive the ratio of damaged cells as explained below (*image processing*).

Cells depolarisation with CCCP. *E. coli* MG1655 suspensions in sample dishes (OD_{600} of 0.05) were treated with 2, 5 or 20 µM CCCP (carbonyl cyanide 3-chlorophenilhydrazone, Fisher Scientific) for 2 hours. During the last 20 minutes, cells were stained with 150 µM $DiOC_2(3)$ (3,3'-Diethyloxacarbocyanine Iodide, Thermo Fisher) and 11 mM EDTA (Ethylenediaminetetraacetic acid, Thermo Fisher) to permeabilise cells membrane permitting ingress of $DiOC_2(3)$. This was done by replacing 3 mL of suspension with the same volume of a staining mixture in M63+ containing the required amount of dye and EDTA. To prevent damage to the sample or device, vibrations were stopped during this step and resumed for the remaining 20 minutes. Vibrational stimulation was then ceased, the samples were diluted 1 in 25 by replacing twice 4 mL of stained suspension with the same volume of fresh M63+ and were finally imaged using fluorescence microscopy. For every condition, 5 to 15 pictures were gathered from the surface in both the red and green channels which were used to determine the ratio of red to green cells. All experiments were performed in triplicate.

Image processing. All image processing protocols were performed using FIJI[10] as either sequencies of manual or automated steps using task specific scripts.

Surface coverage quantification and cells size determination. Fluorescence images (30 - 40 per sample) were analysed using FIJI and the process automated through custom scripts. These determined the

number of cells, their sizes and the fraction of area covered in each picture (Fig. S1). The resulting values were averaged across pictures from three independent replicates and statistically compared. When determining surface coverage of latex beads, the same workflow was adopted. For cells size determination, the same steps above were followed and the resulting cells sizes where averaged across all cells imaged for a given condition.

Surface sedimentation: A sedimentation ratio was defined as the fraction of surface area covered by cells clusters expressed as below:

$$Sedimentation\ Ratio = \frac{Total\ coverage - Cells\ coverage}{Total\ coverage}$$

To determine this, a FIJI script quantified the total surface coverage of cells and clusters (Fig. S2). The same operation was then limited to cell sized objects (4 $\mu m^2 \pm 0.9$) and their coverage determined. For each picture, the resulting value was subtracted from the total to derive the area covered by sediments. This was then divided by the total to obtain the sedimentation ratio in each picture.

Membrane damage from PI staining. Images from both red and green channels were taken as pairs and cells were counted using the same procedure as for surface coverage determination (Fig. S1). For every pair of green and red picture, the ratio of red to green cells was computed. The resulting values among all pictures from three independent replicates were statistically compared between vibrated samples and controls.

Membrane polarisation. (i) *Ratio-metric*: Pairs of red and green images were independently analysed with custom scripts. These quantified the number of red and green cells in each pair and the resulting counts were grouped across pictures from three independent replicates. For each condition, the ratio of red to green counts was used to determine the fraction of polarized over depolarized cells. (ii) *Fluorometric:* Cells fluorescence on the red channel was determined from their pixel intensity. To do this custom FIJI scripts were used to automatically segment the images and isolate single cells from the background. Cells fluorescence was derived as the average pixel intensity of their associated region of interest (ROI). Finally, for a given condition, these values were plotted as histograms, normalised by the total number of cells. This was determined from the grand total of fluorescing cells in the green channel. The resulting distribution were then interpolated using sum of gaussian functions and the fraction of polarised and hyperpolarised cells were determined as the area under the curve respectively below and above 2 standard deviations of the left- most peak of the control distribution.

Parameters estimation for cells motility classification. Recorded videos of cells on surfaces (9 to 12 per condition, 20 s duration) were split into stacks of images that were denoised and compensated for

both potential photo-bleaching and background fluorescence. FIJI's plug-in TrackMate [11,12] was used on the processed stacks to derive cells specific trajectories. To prevent short tracks from biasing the resulting statistics, these were discarded when shorter than 15 s. Cells motility was classified based on their max displacement (maxD) and average body length (δ). The former was derived from cell tracks and the latter from the imaged cells as explained above (Fig. S1). From these values, cells were classified as either *stationary* ($maxD < 0.5\ \delta$), *rotating* ($0.5\ \delta < maxD < 1\ \delta$) or *travelling* ($maxD > 1\ \delta$). The resulting ratios from three independent replicates were then plotted and compared between conditions.

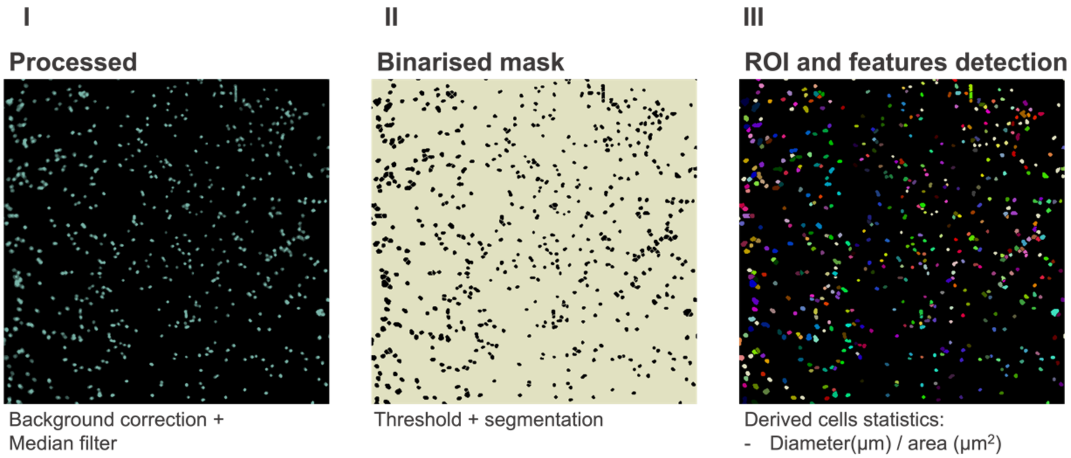

Fig. S1. Schematic representation of image processing for surface coverage and cell size quantification. Fluorescence pictures are corrected for background fluorescence and denoised (I) then a threshold is applied and features segmented (II). Lastly, segmented objects are analysed as individual ROI and desired statistics derived (III).

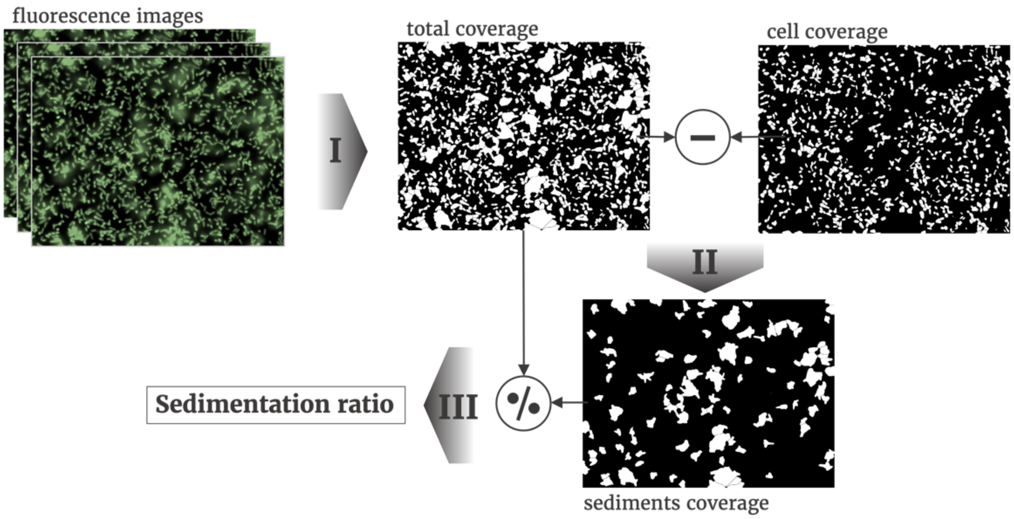

Fig. S2. Image processing steps to determine picture specific sedimentation ratios. Fluorescence images were denoised, background fluorescence removed and pictures binarized (I). The same process was repeated on cell sized objects (4 $\mu m^2 \pm 0.9$). The resulting coverage was subtracted from the total to obtain a sediments coverage (II). This was finally divided by the total to obtain the sedimentation ratio of a given picture.

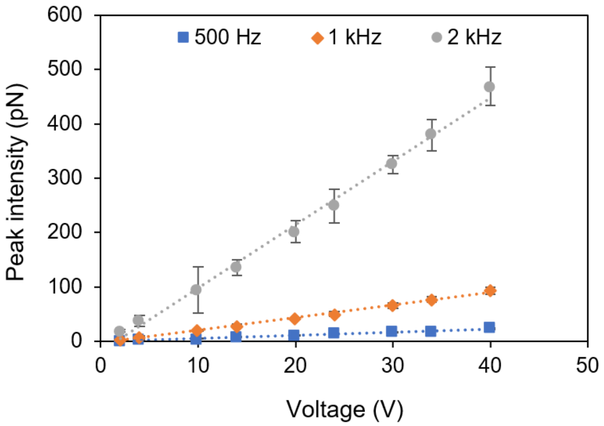

Fig. S3. Modelled peak magnitudes of mechanical forces acting on cells approaching surfaces vibrating at frequencies of 0.5, 1 and 2 kHz under driving potentials of up to 40 V. Values are the average intensity with standard deviation derived from previously determined nanometric vibrational amplitudes as per Eq. 1 (Fig. 1C).

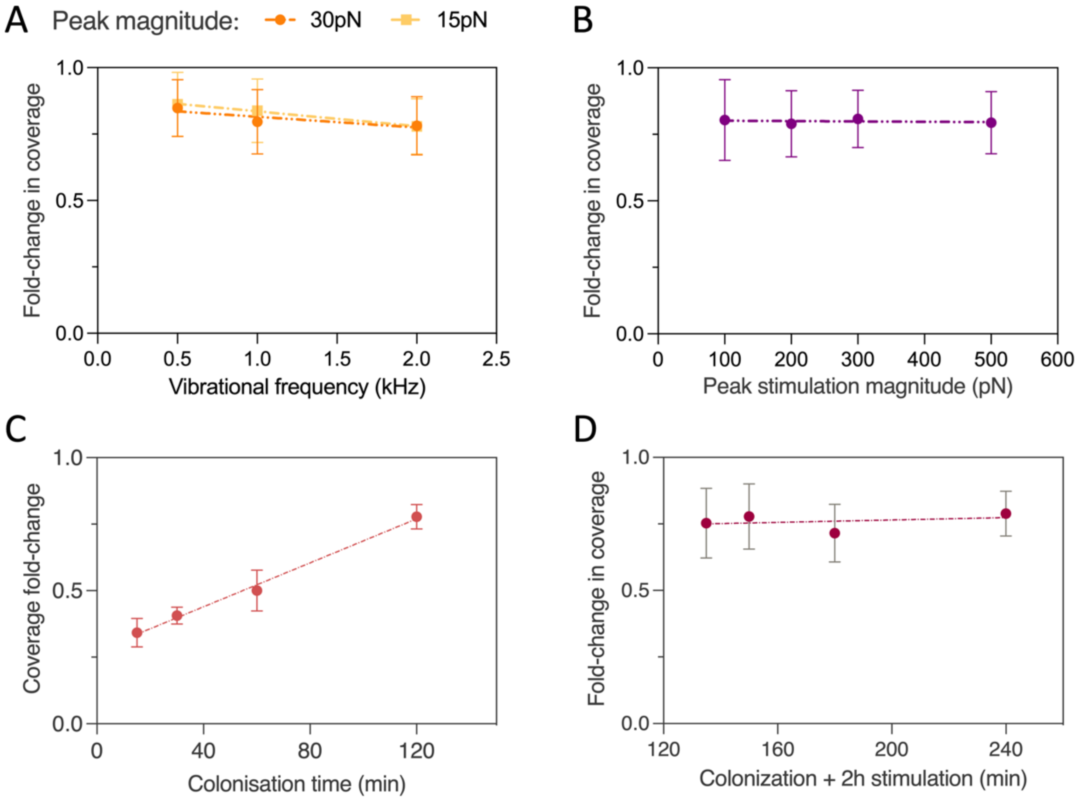

Fig. S4. (A) Fold-change in surface coverage after 2 hours of vibrational stimulation at 15 and 30 pN peak intensity for frequencies between 0.5 and 2 kHz. (B) Fold change in surface coverage after 2 hours on surfaces vibrating at 2 kHz and driving potential generating stimuli of peak magnitude between 100 and 500 pN. The voltages needed to achieve a given intensity were derived from data in Fig. S3 according to Eq. 1. (C) Surface coverage after washing on samples dishes following undisturbed colonization times of 15, 30, 60 and 120 min. Cells concentrations in samples were adjusted as to lead to the same surface coverage before washing. (D) Fold-change in surface coverage after 2 h stimulation at 30 pN (2kHz, 3.7 V) of samples where cells were allowed to pre-colonize the surface for 15, 30, 60 and 120 min before vibrational stimulation. Data on graphs are the average fold change in coverage with standard deviation for microscopy pictures across three biological replicates (n > 95 for all conditions). Lines are linear regressions, $R^2 > 0.81$ for all interpolations.

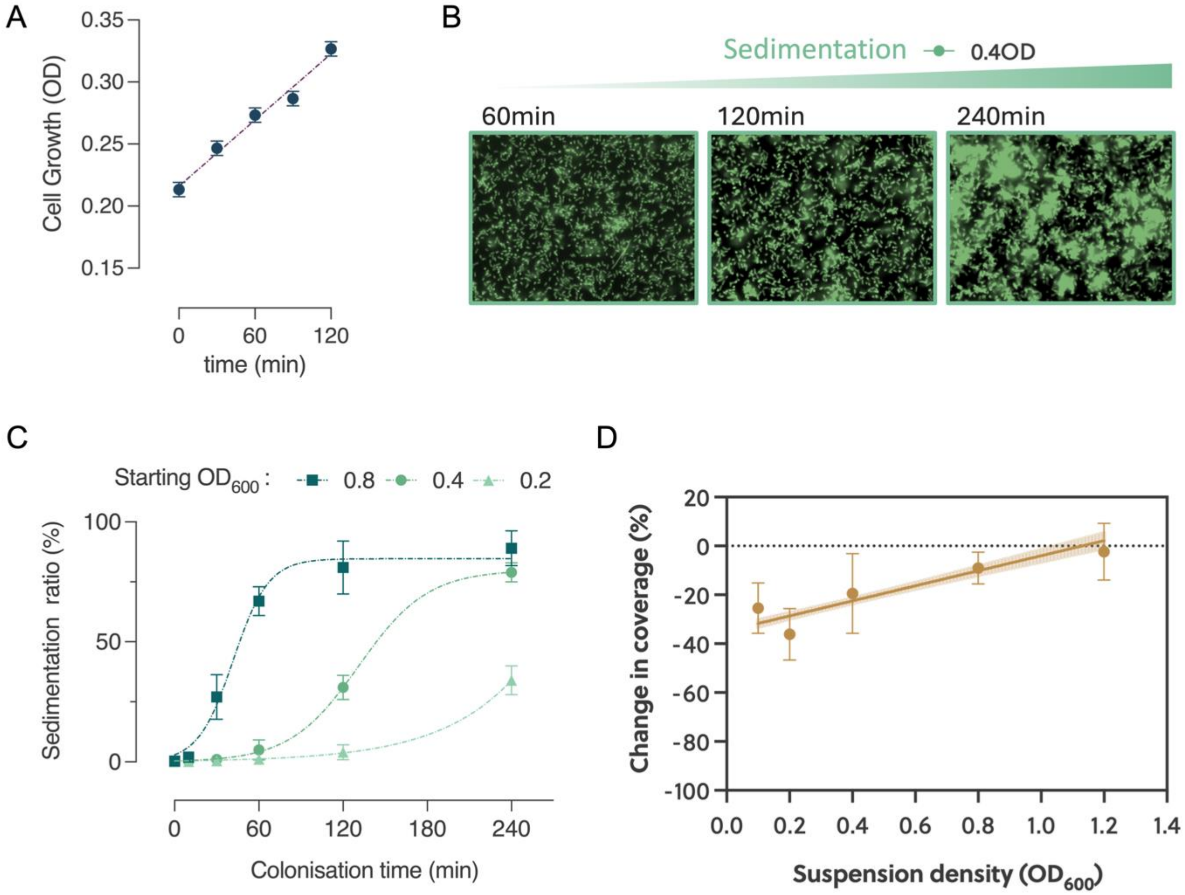

Fig. S5. (A) Increase in OD_{600} following cell growth in M63+ suspensions within sample dishes (35 mm). (B) Fluorescent pictures showing the increase in cells surface sedimentation over time from a starting OD_{600} of 0.4. (C) Sigmoidal growth of surface sediments for starting suspension densities at OD_{600} of 0.2, 0.4 and 0.8. For each condition, datapoints are the mean sedimentation ratios with SD across all pictures of a given time point between three independent replicates ($n > 36$ for all conditions). (D) Change in surface coverage of samples vibrated for 2 hours at 30 pN (2 kHz, 3.7 V) for different starting suspension densities. Lines are sigmoidal and linear interpolation curves.

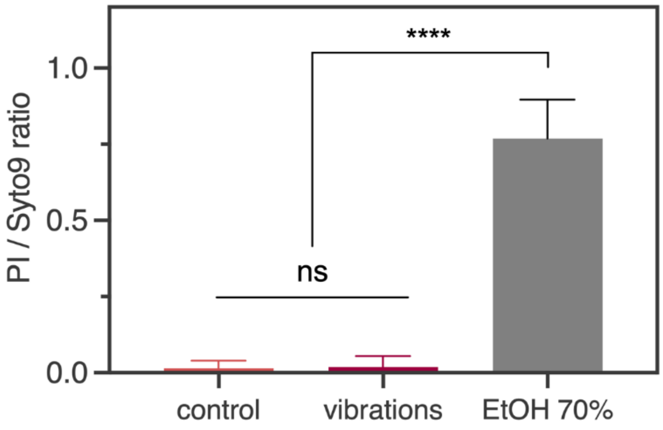

Fig. S6. Fraction of membrane damaged cells on the surface expressed as the ratio of red (propidium iodide, PI stained) to green (healthy, Syto9 stained) *E. coli* SCC1 cells for control and vibrated samples (2 hours, 30 pN, 2 kHz, 3.7 V). On the right, a positive control of stained cells treated with 70 % EtOH for 2 hours. The same PI-stained fractions were observed for vibrated samples and controls (< 0.2 %). Data are the mean and SD of the ratios coming from pictures among three replicates (n > 134 for both control and vibrated samples, ns is for non-significant).

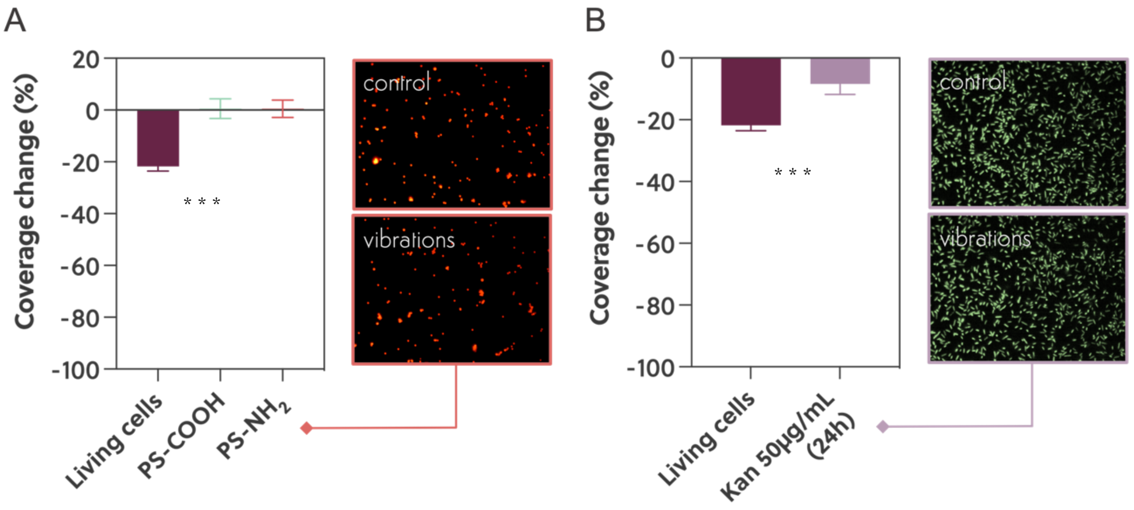

Fig. S7. Change in surface coverage after 2 hours stimulation at 30 pN (2 kHz, 3.7 V) for *E. coli* SCC1, carboxy (PS-COOH) and amine (PS-NH_2) functionalized fluorescent latex beads (A) and kanamycin treated cells (B, 24 hours, 50 µg/mL). Data are the mean change in coverage with 95 % CI (n > 130 for all conditions, $p^{****} < 0.0001$). Inserts are fluorescence pictures from the surface representing amine functionalized beads (A) and dead cells (B) on both control and vibrated samples.

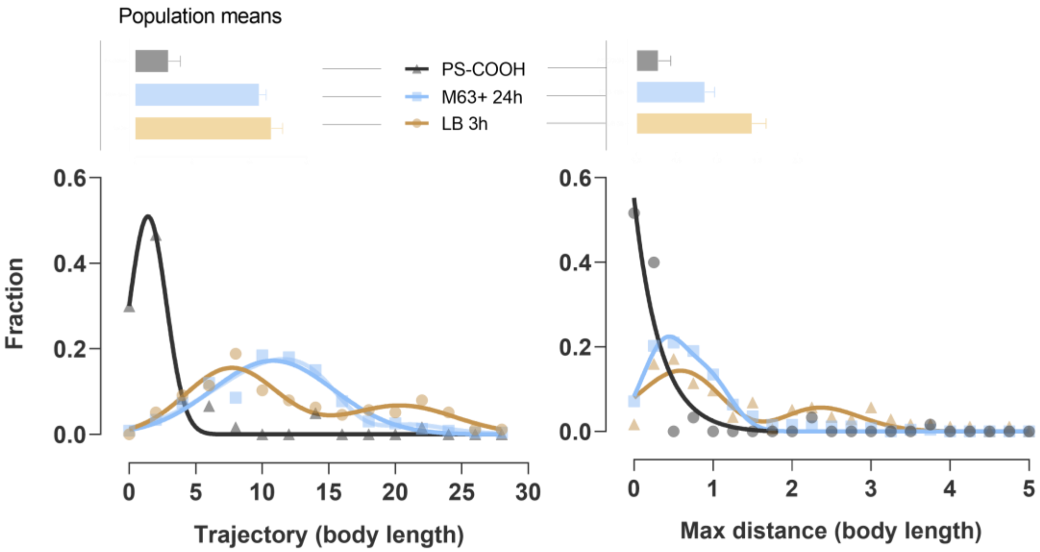

Fig. S8. Population averaged and underlying frequency distributions of both the total track length (left) and max displacement (right) for tracked polystyrene carboxy functionalised beads (PS-COOH) and slow and fast-growing cells in respectively minimal M63+ or rich LB media. Bars plots show the mean and 95 % confidence intervals of the total tracked objects per conditions (n = 60, 233 and 175 for PS-COOH, M63+ and LB respectively). Solid lines are gaussian and exponential interpolations ($R^2 > 0.85$ for all conditions).

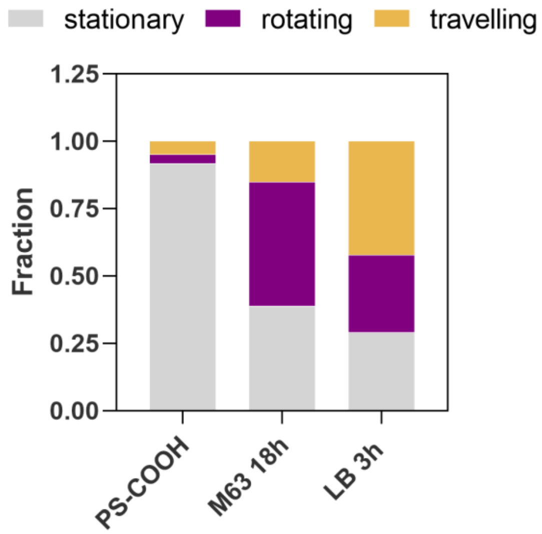

Fig. S9. Fraction of stationary, rotating and travelling objects on polystyrene surfaces for polystyrene carboxy-functionalised beads (PS-COOH), slow-growing cells in M63+ minimal medium and fast-growing cells in LB medium (n = 60, 233 and 175 for PS-COOH, M63+ and LB respectively).

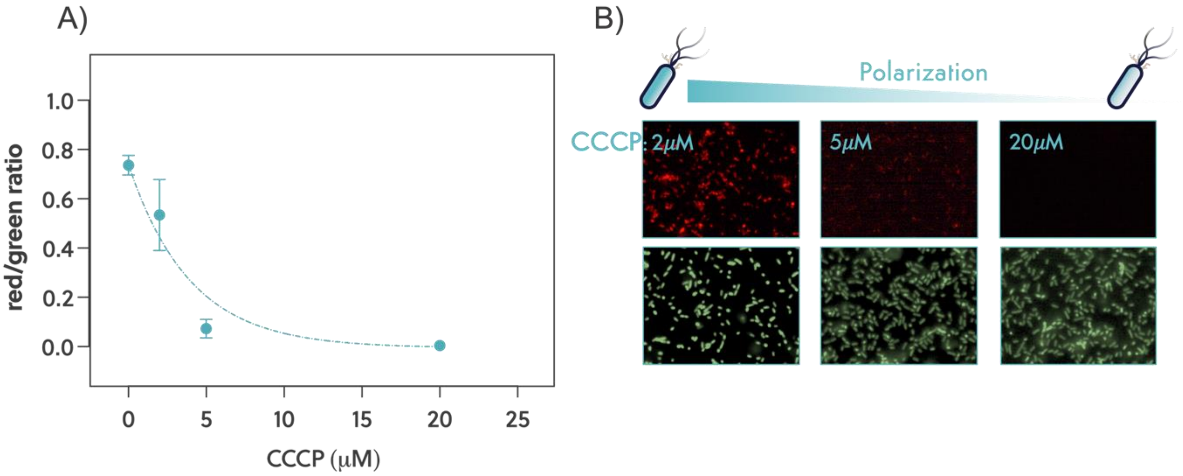

Fig. S10. Depolarising effect of CCCP on cells membrane potential. (A) Fraction of polarised cells (red) over total (green) decreases with increasing CCCP concentrations. Data are the mean and SD of the resulting ratios from all pictures across three independent replicates. Dashed line is an exponential decay interpolation. (B) Fluorescence images from surface attached cells stained with 150 μM $DiOC_2(3)$ and 11 mM EDTA. The number of polarised (red) cells decrease with increasing CCCP concentrations.

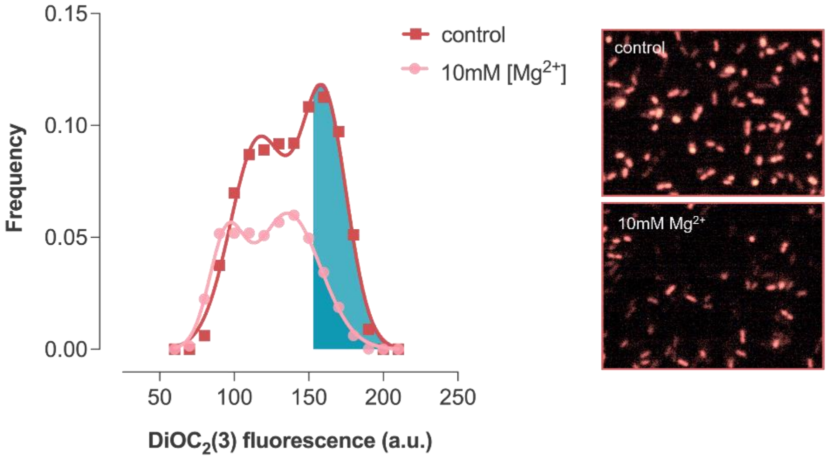

Fig. S11. Frequency distribution of cells membrane potential ($DiOC_2(3)$ fluorescence at 690 nm) after 2 hours treatment with 10 mM Mg^{2+} ($n > 10^3$ and $> 10^4$ for 10 mM Mg^{2+} and control respectively). Shaded area is the area under the sum of gaussian interpolating curve taken beyond 2 SD of the leftmost peak in control samples ($R^2 = 0.98$). Insert on the right are red fluorescing cells imaged on the surface.

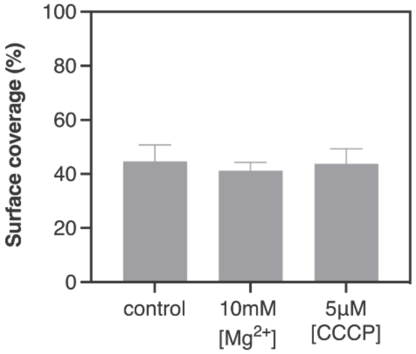

Fig. S12. Surface coverage on polystyrene petri dishes after 2 hours. To achieve the same coverage as control samples, starting suspension densities for cells treated with Mg^{2+} and CCCP were respectively increased to OD values of 0.27 and 0.33 (n > 108 per condition).

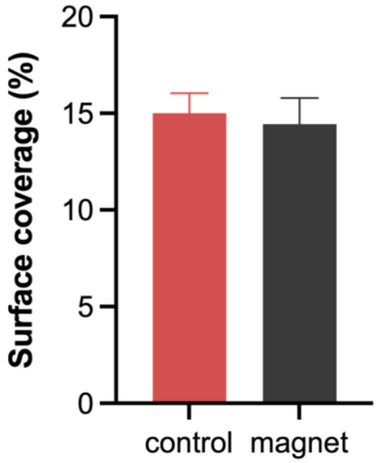

Fig. S13. *E. coli* SCC1 surface coverage on polystyrene petri dishes after 1 hours of being placed on a neodymium magnet (starting suspension density was set at OD_{600} of 0.4). Data are the mean and SD of surface coverage from fluorescence pictures across three independent replicates (n > 121 per condition).

References:

1. Beloin, C., Roux, A. & Ghigo, J. M. Escherichia coli biofilms. *Curr Top Microbiol Immunol* **322**, 249–289 (2008).
2. Barnhart, M. M. & Chapman, M. R. Curli Biogenesis and Function. *Annual Review of Microbiology* **60**, 131–147 (2006).
3. Keseler, I. M. *et al.* EcoCyc: a comprehensive database of Escherichia coli biology. *Nucleic Acids Res* **39**, D583-590 (2011).
4. Andreassen, P. R. *et al.* sRNA-dependent control of curli biosynthesis in Escherichia coli: McaS directs endonucleolytic cleavage of csgD mRNA. *Nucleic Acids Res* **46**, 6746–6760 (2018).
5. Duncan, M. J. *et al.* The Distinct Binding Specificities Exhibited by Enterobacterial Type 1 Fimbriae Are Determined by Their Fimbrial Shafts*. *Journal of Biological Chemistry* **280**, 37707–37716 (2005).
6. Danese, P. N., Pratt, L. A., Dove, S. L. & Kolter, R. The outer membrane protein, antigen 43, mediates cell-to-cell interactions within Escherichia coli biofilms. *Mol Microbiol* **37**, 424–432 (2000).
7. Tagliabue, L. *et al.* The diguanylate cyclase YddV controls production of the exopolysaccharide poly-N-acetylglucosamine (PNAG) through regulation of the PNAG biosynthetic pgaABCD operon. *Microbiology* **156**, 2901–2911 (2010).
8. Sande, C. & Whitfield, C. Capsules and Extracellular Polysaccharides in Escherichia coli and Salmonella. *EcoSal Plus* **9**, eESP00332020 (2021).
9. Miao, H., Ratnasingam, S., Pu, C. S., Desai, M. M. & Sze, C. C. Dual fluorescence system for flow cytometric analysis of Escherichia coli transcriptional response in multi-species context. *Journal of Microbiological Methods* **76**, 109–119 (2009).
10. Schindelin, J. *et al.* Fiji: an open-source platform for biological-image analysis. *Nat Methods* **9**, 676–682 (2012).

11. Ershov, D. *et al.* TrackMate 7: integrating state-of-the-art segmentation algorithms into tracking pipelines. *Nat Methods* **19**, 829–832 (2022).

12. Tinevez, J.-Y. *et al.* TrackMate: An open and extensible platform for single-particle tracking. *Methods* **115**, 80–90 (2017).
